# Poor newborn care practices - a population based survey in eastern Uganda

**DOI:** 10.1186/1471-2393-10-9

**Published:** 2010-02-23

**Authors:** Peter Waiswa, Stefan Peterson, Goran Tomson, George W Pariyo

**Affiliations:** 1 Makerere University School of Public Health, Kampala, Uganda; 2 Global Health, Department of Public Health Sciences, Karolinska Institutet, Stockholm, Sweden; 3 International Maternal and Child Health, Department of Women's and Children's Health, Uppsala University, Uppsala, Sweden; 4 Medical Management Centre, Karolinska Institutet, Stockholm, Sweden

## Abstract

**Background:**

Four million neonatal deaths are estimated to occur each year and almost all in low income countries, especially among the poorest. There is a paucity of data on newborn health from sub-Saharan Africa and few studies have assessed inequity in uptake of newborn care practices. We assessed socioeconomic differences in use of newborn care practices in order to inform policy and programming in Uganda.

**Methods:**

All mothers with infants aged 1-4 months (n = 414) in a Demographic Surveillance Site were interviewed. Households were stratified into quintiles of socioeconomic status (SES). Three composite outcomes (good neonatal feeding, good cord care, and optimal thermal care) were created by combining related individual practices from a list of twelve antenatal/essential newborn care practices. Multiple logistic regression analysis was used to identify determinants of each dichotomised composite outcome.

**Results:**

There were low levels of coverage of newborn care practices among both the poorest and the least poor. SES and place of birth were not associated with any of the composite newborn care practices. Of newborns, 46% had a facility delivery and only 38% were judged to have had good cord care, 42% optimal thermal care, and 57% were considered to have had adequate neonatal feeding. Mothers were putting powder on the cord; using a bottle to feed the baby; and mixing/replacing breast milk with various substitutes. Multiparous mothers were less likely to have safe cord practices (OR 0.5, CI 0.3 - 0.9) as were mothers whose labour began at night (OR 0.6, CI 0.4 - 0.9).

**Conclusion:**

Newborn care practices in this setting are low and do not differ much by socioeconomic group. Despite being established policy, most neonatal interventions are not reaching newborns, suggesting a "policy-to-practice gap". To improve newborn survival, newborn care should be integrated into the current maternal and child interventions, and should be implemented at both community and health facility level as part of a universal coverage strategy.

## Background

In low income countries (LICs), progress towards achieving Millennium Development Goal 4 - to reduce by two-thirds under-5 mortality from the 1990 baseline - is being hampered by slow progress in reducing neonatal death [1]. The neonatal period is only 1/60 of the first five years of life, but contributes 38% of the estimated 10.5 million under-five deaths which occur every year [2]. It is estimated that each year four million neonatal deaths occur, and almost exclusively in low income countries [3]. There is a paucity of data on newborn health from sub-Saharan Africa, and few studies have assessed inequity in uptake of newborn care practices.

The World Health Organisation recommends improving care practices at birth in order to reduce neonatal morbidity and mortality. These have been described as essential newborn care (ENC) practices [4] and include clean cord care, thermal care and initiating breast feeding immediately or within the first hour after birth. These simple practices are critical for all babies in order to save lives, but also need to be fitted into a comprehensive newborn care package which includes skilled care at birth, care-seeking, extra care for sick and small babies, and skilled care at birth including resuscitation. Effective promotion of ENC at scale could significantly contribute to reducing the leading causes of newborn deaths in LICs, especially those due to sepsis/pneumonia, preterm births and tetanus [5].

To be effective, such evidence-based interventions need to be implemented within the continuum of care for maternal, newborn and child (MNC) care [5-7]. The continuum of care for MNC health has been defined as "access to care provided by families and communities, by outpatient and outreach services, and by clinical services throughout the lifecycle, including adolescence, pregnancy, childbirth, the postnatal period, and childhood [5-7]. An effective continuum implies that care of the mothers and babies at home, in lower level facilities, and in referral centres is adequately linked and is of high quality [5-7]. From a conceptual point of view, this is the ideal strategy, as its implementation would benefit both mothers and newborn babies [5]. However, given the high maternal and newborn mortality in sub-Saharan Africa, it is clear that there is either no adequate quantity and/or quality in the MNC continuum in these countries or it may be suggestive of the difficulties in its implementation.

Maternal and newborn outcomes remain very poor in Uganda. The total fertility rate is 6.7, while the under five mortality rate is 137/1000, the neonatal mortality rate 29/1000, and maternal mortality ratio is 435/100,000 live births [8]. Antenatal care attendance at least 4 times is 42% and only 41% of deliveries are supervised. These poor indicators are despite the fact that ANC attendance at least once during pregnancy is at 92% [8] and 72% of the population is within 5 km distance of a health facility [9]. Although recommended in policy, in practice virtually no post-natal care exists.

Inequity in child health is high in Africa but few studies have assessed it with respect to newborn care. Mortality is higher and coverage is lower among the poorest, implying that addressing the needs of the poorest should be a priority for all programmes [10]. Analysing mortality and coverage by SES provides information that is hidden in national and even regional averages. It has been argued that when packaged, child survival interventions are less equitable, with children belonging to the poorest families consistently being less likely to receive preventive and curative interventions [11]. The aim of this study was to assess socioeconomic differences in levels of coverage of essential newborn care practices in order to inform programming and policy in Uganda.

## Methods

### Study setting

The study was conducted in the Makerere University-operated Iganga-Mayuge Demographic Surveillance Site (DSS) located in eastern Uganda, about 120 km east of the capital Kampala. The *Basoga *contribute about 10% of the population of Uganda, but their practices are similar to those of other Bantu ethnic groups who are the majority in Uganda. Eighty percent of the population are peasants and live on less than US$1 a day. An estimated 49% of women and 68% of men are literate (Iganga District Local Government 2008). Traditional birth attendants (TBAs) are significant actors in the provision of antenatal and delivery care in the district. At the time of the study, there were no specific interventions promoted to target the newborn, either at facility or community level. About 30% of the DSS population lives in peri-urban settings with relatively better access to health care compared to their rural counterparts.

The DSS has a population of about 67,200 people in 65 villages, 18 parishes and 12,000 households. The household and community structures have been mapped using the Global Positioning System. Over forty locally recruited field assistants whose minimum education is upper secondary school level collect data from each household every fourth month and are supervised by a group of DSS staff from a central office. Village-based demographic scouts notify DSS staff of all deaths and births in the area as they occur on a continuous basis. The DSS area has 13 health facilities of which ten are government facilities including the district hospital, the other three being non-Governmental organisation facilities. The area is also served by over 120 pharmacies and private clinics. The neonatal and post-neonatal mortality rates in the DSS are estimated at 22.3 and 55.2 per 1000 live births, which compares very well with estimates for the entire region as reported in the national demographic health survey (24 and 50 per 1000 live births) [8].

### Study design and data collection

This population-based cross-sectional study represents socio-demographic, SES, and antenatal and newborn care practices among Ugandan women with a baby aged 1-4 months (n = 414). Socio-demographic and household SES information were collected in a separate survey a year earlier. Socio-demographic information, as collected from the DSS, included age, level of education, occupation, religion, tribe, birth order, and sex of the reference child. Household SES is represented by household assets.

The DSS field assistants underwent a three day training to use the survey tool which had been translated into *Lusoga*, the local language in the area. The training included piloting the tool among 25 mothers attending a postnatal clinic at the local hospital. The survey was conducted from March to August 2007. Mothers who had had a stillbirth (data not available) or a neonatal death (64 neonates) were not interviewed for this study. Data collected in this study included information about antenatal care (ANC) practices (attendance, place of attendance, number of visits made, HIV testing, birth preparedness, use of drugs to prevent malaria in pregnancy, and provider of ANC) and delivery (place, time of labour onset and type of attendant at delivery). Women were also asked about their experiences with ENC practices, including type of instrument used to cut the cord, type of material used to tie the cord, when the newborn was first dried and wrapped, length of time before the newborn was bathed the first time, whether any pre-lacteal feeds were given, length of time (hours/days) before breastfeeding was first initiated, and whether the baby was exclusively breastfed during the first month of life. The quality assurance of data was through daily assessment via questionnaires filled-in by a supervisor; in cases of error or incompleteness of data, corrective measures were implemented immediately.

### Statistical analysis

Data were entered using FoxPro and cleaned, linked with the DSS database, and then transferred to STATA, version 10, for analysis. For SES, we used the same group of context-specific assets used by the Uganda Bureau of Statistics. These items were screened for relevance, and reliability testing was done using Cronbach's alpha [12]. The final list included the number of sleeping rooms, type of floor material, type of roof material, wall material, fuel used for cooking and source of light. Other variables were households having or not having the following items: a radio, a sewing machine, an electric flat iron, type of bed, charcoal flat iron, a bed net, kerosene lamp, kerosene stove, car, tea table, refrigerator, television set, sound stereo, telephone, mattress, wheel barrow, cell phone and camera. These gave a Cronbach's alpha of 0.848. Principal component analysis (PCA) was performed and the first principal component was scored to create an asset index that was used to group all households in the DSS into wealth quintiles [13].

Using the following twelve ANC/ENC practices, we calculated the mean and median number of practices accessed by the mother/newborn: ANC, tetanus toxoid, antimalarial use during pregnancy, HIV test, and insecticide treated net (ITN) use, anemia drugs, clean birth, facility delivery, safe cord care, optimal thermal care, good breastfeeding, and ITN after birth. The following composite outcome variables were then created: (i) Good cord care (defined as use of a clean cutting instrument to cut the umbilical cord plus clean thread to tie the cord plus no substance applied to the cord); (ii) Optimal thermal care (defined as baby put skin-to-skin at birth or wrapped at birth plus first bath after 6 or more hours); and (iii) Good neonatal breastfeeding (defined as initiating breastfeeding within the first one hour after birth plus baby given no supplements at all in the first month of life). These composite variables were then dichotomised to Yes (all practices present) or No (one or more practices missing).

The data were then subjected to standard descriptive analysis. Chi-square statistics were performed to compare the levels of each of the dependent variables with the explanatory variables. A multiple logistic regression model was constructed for each dichotomised outcome variable using all of the explanatory variables which were significant at bivariate analysis at a p-value of 0.05 or less after confirming absence of multi-colleneraity between the independent variables.

The study was approved following ethical review by the Makerere University School of Public Health Institutional Review Board. As per the DSS routines for non-intrusive research verbal consent was sought from each mother after reading to her about and adequately explaining the purpose of the study. Participants were told that they were free not to participate or to withdraw during any stage of the interview. In addition, field assistants were trained to refer sick mothers/newborn babies with problems to the nearby government health facilities where treatment is provided free of charge.

## Results

Of the mothers interviewed about neonatal care practices, only 393 had SES data provided from the previous year's survey. Most of the mothers were young, with 44% being 25 years or below, and only 27% were above 30 years (Table 1). The majority of respondents were of the Ba*soga *tribe, were married or living together, and almost three-quarters had only primary or no education. Three-quarters of the mothers were multiparous.

**Table 1:** Baseline characteristics of the respondents

Characteristics	Total	%
**Maternal Age n = 393**		
<19	35	9
19 - 25	139	35
26 - 30	114	29
>30	105	27
**Marital status n = 321**		
Married/Living together	291	91
Not married	30	9
**Education n = 310**		
No education	39	13
Primary	208	67
Secondary	63	20
**Socioeconomic group n = 340**		
1 (Lowest)	51	15
2	67	20
3	87	26
4	84	25
5 (Highest)	51	15
**Parity n = 393**		
1	97	25
2-4	137	35
> 4	159	40
**No. of ANC visits n = 377**		
1	35	9
2-3	225	60
>3	117	31
**Trimester of 1^st ^ANC visit n = 278**		
1	50	13
2	207	55
3	121	32

**Distance to place of delivery n = 390**		
None	129	33
< 5 km	200	51
> 5 km	61	16

### Levels of coverage of essential newborn care practices

In half the respondents, labour and delivery occurred at night. In general, there were low levels of coverage of the desired practices (Table 2). A total of 46% of the respondents delivered in the hospital or in a health unit, 26% delivered in private clinics and 28% at home or with TBAs. Cord cutting was mostly by use of a razorblade (67%) of which 10% were reused, and only 28% reported to have used cord scissors. About half of the mothers put substances on the cord (such as powder, surgical spirit, salty water, or lizard droppings).

**Table 2:** Level of selected care practices during delivery and neonatal period

Characteristics	Total	%
**Time labour began n = 356**		
Day	146	42
Night	205	58
**Time of delivery n = 391**		
Day	195	50
Night	196	50
**Health facility delivery n = 393**		
**Yes**	181	46
**No**	212	54
**Surface of delivery n = 392**		
Clean	258	66
Dirty	134	34
**Instrument used to cut the cord n = 391**		
Clean	333	85
Not clean	58	15
**Material used to tie the cord n = 391**		
Clean	387	99
Not clean	4	14
**Type of instrument used to cut the cord n = 391**		
Un used new razor blade	223	57
Used razor blade	41	10
Scissors	110	28
Other/Don't know	17	5.0
**What was used to tie the cord n = 391**		
Cloth strip	39	10.0
Clean thread	338	86.4
Rubber band	3	0.1
Other/Don't know	11	2.8
**What was put on the cord n = 389**		
Nothing	198	51
Medical drugs	11	3.0
Powder	87	22.2
Ash	3	0.8
Salty water	43	11
Other	47	12
**How long after birth baby was breastfed n = 392**		
Immediately	199	51
Less than 6 hours	159	41
6 - 24 hours	24	6.0
>24 hours	10	2.0
**If at all, bottle fed in neonatal period n = 391**		
Yes	42	11
No	349	89
**How long after birth was baby first bathed n = 244**		
Less than 1 hour	13	5
2- 6 hours	125	51
7 - 12 hours	63	26
13 - 24 hours	34	14
>24 hours	9	4
**Safe cord care n = 387**		
Yes	149	39
No	238	61
**Good neonatal feeding n = 378**		
Yes	216	57
No	162	43
**Optimal thermal care n = 398**		
Yes	166	42
No	226	58

To keep the babies warm, 86% were immediately wrapped, but skin-to-skin (STS) care was almost non-existent (2%). Early bathing was the norm, with 56% of the babies bathed within the first 6 hours, 82% within the first 12 hours and almost all during the first 24 hours. Although all babies were breastfed, only about half were initiated within the first hour of birth, with 41% initiating within 1 - 6 hours. Other feeds besides breast milk, including cow's milk, plain water, sugar or glucose water, gripe water and tea, were given to 35% of babies in the neonatal period, contrary to recommendations.

Of the twelve practices assessed, we found that neonates in the upper quintile received more interventions/practices than their counterparts (test for trend p < 0.001). The mean number of practices received was 7.3 (IQR 6-9).

### Coverage of newborn interventions across socioeconomic groups

There was a low level of use of the composite newborn care indices of safe cord care (38%), good neonatal feeding (57%) and optimal thermal care (42%). We found that poor cord care was driven mainly by putting substances on the cord; poor thermal care by early bathing and no STS practice; and poor breastfeeding by giving feeds other than breast milk (results not shown).

### Predictors of essential newborn care practices

Surprisingly, SES was not an independent predictor of any of the composite newborn care practices assessed (Table 3). In bivariate comparisons, significant differences in levels (*x*^2 ^*Prob < 0.05) *for each of the three outcomes were observed only for clean cord care: parity and the time at which labour had begun. There was no significant association between any of the explanatory variables (including ANC) with either optimal thermal care or good neonatal feeding.

**Table 3:** Levels of selected newborn care practices by socio-demographic and antenatal and delivery care factors

Characteristic	Safe cord care %, p-value		Thermal care %, p-value		Neonatal feeding %, p-value	
**Maternal Age**						
<19	11		8		8	0.60
19-25	32	0.04	39	0.63	38	
26-30	24		26		29	
>30	33		27		24	
**Parity**						
1	32	0.01	25	0.77	25	0.81
2-4	27		33		37	
>4	42		42		39	
**Social group**						
1(Lowest)	14		15		13	0.58
2	22	0.42	23	0.66	19	
3	24		27		26	
4	28		22		25	
5 (Highest)	12		13		18	
**ANC attendance**						
1	10	0.72	11	0.32	8	0.82
2-3	62		55		61	
>3	28		34		31	
**Time of 1st visit**						
1^st ^trimester	11	0.59	12	0.97	13	0.97
2^nd ^trimester	55		55		55	
3^rd ^trimester	34		33		32	
**Tested for HIV**						
Yes	43	0.71	39	0.49	38	0.13
No	57		61		62	
**Time labour began**						
Day	53	0.06	44	0.22	47	0.56
Night	47		56		53	
**Time of delivery**						
Day	50	0.98	48	0.44	49	0.80
Night	50		52		51	
**Distance to clinic**						
None	28	0.36	35	0.78	33	0.82
<5 Km	56		49		52	
5+ Km	16		16		15	
**Birth prepared**						
Yes	87	0.67	84	0.17	86	0.79
No	13		16		14	
**Pregnancy counselling**						
Yes	96	0.64	94	0.26	95	0.19
No	4		6		5	

Table 4 shows the independent predictors of safe cord care. Multiparous mothers were less likely to have good cord practices when compared to primiparous (OR 0.5, CI 0.3 - 0.9); and so were mothers whose labour began at night compared to those whose labour began during day time (OR 0.6, CI 0.4 - 0.9). Although significantly more mothers with high SES delivered in health facilities (p < 000; results not shown), we found that place of delivery did not predict any of the ENC practices assessed.

**Table 4:** Logistic models with safe cord care practices as dependent variable versus all independent variables that had significant chi-square values in bivariate analysis

Variable	Univariate Unadjusted		Multivariate Adjusted*	
	OR	95% CI	OR	95% CI
**Maternal Age**				
<19	1		1	
19-25	0.52	0.24-1.11	0.68	0.31-1.51
26-30	0.47	0.21-1.03	0.62	0.26-1.47
>30	0.89	0.41-1.93	1.19	0.48-2.95
**Parity**				
1	1			
2-4	0.44	0.25-0.76	0.45	0.25-0.79
>4	0.68	0.40-1.13	0.57	0.30-1.08
**Time labour began**				
Day	1			
Night	0.66	0.44-1.01	0.61	0.4. - 0.94

*Adjusted for Maternal age, parity and time labour began

* p for the whole model = 0.003

## Discussion

The findings, drawn from a population-based survey of all mothers of infants in a demographic surveillance site in two rural districts of Uganda, show that the proportion of newborn babies who receive the essential newborn care practices is generally low and does not differ much by socioeconomic group. We were expecting the newborn practices of mothers of higher SES to be better than those of their counterparts of lower SES, but we found no difference, which was surprising. In addition, none of the explanatory variables we assessed predicted any of the assessed composite newborn outcomes. A study in rural India reported that use of antenatal care and skilled attendance at delivery were significantly associated with clean cord care and early breastfeeding, but not with thermal care [14].

We found that the low level of safe cord care was due mainly to putting substances on the cord, that poor thermal care was mainly due to early bathing, and that poor breastfeeding was primarily due to giving feeds other than breast milk. Although not recommended, about half the respondents applied substances such as powder, salty water, and lizard droppings to the umbilical cord. Clean cord care is very important in preventing early neonatal infections [5]. We thus found cord care to be wanting. In the absence of supplies in health facilities, cord cutting was done mainly with razorblades, some of which were not clean - a dangerous practice for both mother and newborn given especially that tetanus is still a problem in this area [15] as well as the high prevalence of HIV in pregnancy (6.5%) in Uganda [16]. Mothers apply substances to the cord for several reasons, including to "help the cord heal fast" so that they can go back to their domestic chores [17]. Similar findings have been reported from studies elsewhere in Uganda [18] and in Tanzania [19].

Regarding prevention of hypothermia, we found that STS care was generally not practiced. Currently, STS care is recommended for all babies [20]. In addition, whereas it is recommended that babies should be bathed no earlier than 24 hours after birth, we found instead that most were bathed within the first 12 hours, and almost all within 24 hours of birth. Maintaining good thermal care at birth is crucial for preventing hypothermia, hypoglycemia and neonatal infections. Indeed, studies in Uganda have shown that even if it is a tropical country, hypothermia at birth is common [21,22].

Our findings indicate that although almost all mothers breastfed their babies, about half of the infants were not breastfed within the first one hour as is recommended [23], thereby putting these neonates at an increased risk for death [24]. In addition, more than one-third of respondents reported that they gave feeds other than breast milk in the neonatal period. A study by Engebretsen et al. conducted in eastern Uganda [25] found that only 7% of infants were exclusively breastfed by age three months. In other words, both her study and ours show that as early as the neonatal period, over one-third of infants are not having exclusive breastfeeding.

The proportion of newborn babies receiving the recommended ENC practices was low but similar among both the poor and the least poor. The finding that there is no inequity in newborn care practices was not expected, and we found no literature describing it. Usually, access to childhood interventions is lowest among the poorest and highest among the least poor [11]. Many studies have described inequities in access to childhood interventions [11,26,27]. However, we found only a few studies which have described inequities in newborn health [28,29]. In a study in rural India, Baqui et al. examined whether NGO facilitation of the government's community-based health programme improved the equity of maternal and newborn health and found that improvement in equity was most pronounced in household practices, but inequity was still marked in health care utilisation [29]. In another study, Fenn and colleagues assessed within-country inequities of neonatal mortality and coverage of key interventions using wealth quintiles in eight low income countries [28]. They found that access to interventions was directly related to socioeconomic level, with poorer mothers having reduced access to interventions compared with those in the wealthier groups. However, their findings are based on individual practices whose coverage may be high; and yet when combined into a composite measure may be quite low, as demonstrated in this study.

We hypothesise that our findings may have another explanation. Due to increasing socioeconomic development, a number of mothers are putting powder on the cord, using a bottle to feed the baby, and mixing/replacing breastfeeding with various substitutes. We describe this phenomenon as "modernistic" and suggest that there may be a newborn "practice transition" to practices wrongly perceived to be safe. The Uganda Demographic Health Survey [8] and a study in western Uganda found that educated mothers were more prone to use pre-lacteals [30]. Other studies have also demonstrated that socioeconomic status has little effect on neonatal practices such as breastfeeding practices [31].

Why are these newborn care practices low across SES? We have two possible explanations: 1. Since newborn care is a neglected area, there are currently no effective programmes promoting newborn care practices either in health facilities or at community level, and the low proportion of newborn babies with appropriate newborn care practices could thus be a case of lack of adequate knowledge and exposure to messages [30]; 2. It could be that the population we studied is relatively homogenous and the absolute differences are generally not significant between the upper and lower quintiles [32], (e.g. most women had only primary education).

These findings have important programme and policy implications if neonatal health is to be improved in Uganda and other low income countries within the context of changing demographics, including urbanisation. Since the ENC practices were inadequate among both the poor and least poor, to ensure equity programme implementation should focus on universal coverage and not simply target the poorest members of the community [33]. In addition, the possibility of change in newborn care practices needs to be watched, including aspects of safety to a newborn baby. Finally, we recommend that evaluation of newborn care practices should include both the individual but also the composite practices. Coverage of individual practices may be high, and yet when combined into a composite measure may be quite low.

The results presented here should be interpreted with some caution. The survey was done in only one region of Uganda. However, the study setting did have relatively good physical access to health facilities than would be expected for most areas of rural Uganda, meaning that newborn care practices elsewhere may not be much better than those reported here. In addition, we were not able to verify the actual practices as data were collected through recall, and we excluded mothers who had a stillbirth or a newborn that had died. To minimise recall bias, we limited our interviews to only mothers with babies up to four months. Similar studies report recall up to one year after birth [14,34].

## Conclusions

Both demand and supply side strategies are needed to ensure that these simple but essential interventions are universally promoted within the continuum of care at scale irrespective to socioeconomic status in order to improve newborn survival in Uganda and in similar settings. The level of coverage of essential newborn care practices in this setting is generally poor and does not differ by socioeconomic grouping when assessed as composite practices. Despite being policy, most neonatal interventions are not reaching newborns, suggesting a "policy-to-practice gap". A newborn care "practice transition" in which "suboptimal" practices are being replaced with "modernistic" practices may be underway, requiring attention and action.

## Competing interests

The authors declare that they have no competing interests.

## Authors' contributions

PW, SP, GT and GWP conceived the study. PW, SP, GT and GWP took part in designing the study, in tool development, analysis and manuscript writing. PW supervised the field work, and led the analysis and drafting of the manuscript. All authors approved the final manuscript.

## Pre-publication history

The pre-publication history for this paper can be accessed here:

http://www.biomedcentral.com/1471-2393/10/9/prepub
